# Contemporary Trends and Risk Factors of Hemodynamic and Myocardial Mechanics Derived by the Pressure Recording Analytical Method After Pediatric Cardiopulmonary Bypass

**DOI:** 10.3389/fcvm.2021.687150

**Published:** 2021-07-20

**Authors:** Xiaobin Lou, Yingying Liu, Yanqin Cui, Jianbin Li, Lijuan Li, Li Ma, Minghui Zou, Xinxin Chen, Jia Li

**Affiliations:** ^1^Guangdong Provincial Key Laboratory of Research in Structural Birth Defect Disease, Guangzhou Women and Children's Medical Center, Guangzhou Medical University, Guangzhou, China; ^2^Clinical Physiology Laboratory, Institute of Pediatrics, Guangzhou Women and Children's Medical Center, Guangzhou Medical University, Guangzhou, China; ^3^Cardiac Intensive Care Unit, Heart Center, Guangzhou Women and Children's Medical Center, Guangzhou Medical University, Guangzhou, China; ^4^Cardiovascular Surgery, Heart Center, Guangzhou Women and Children's Medical Center, Guangzhou Medical University, Guangzhou, China

**Keywords:** pressure recording analytical method, systemic hemodynamics, cardiac cycle efficiency, epinephrine, pediatric cardiopulmonary bypass, deep hypothermic circulatory arrest

## Abstract

**Objective:** Adverse factors of postoperative hemodynamic and myocardial performance remain largely unexplored in children with congenital heart disease following cardiopulmonary bypass due to technical limitations. Pressure recording analytical method (PRAM) is a continuous hemodynamic and myocardial performance monitoring technique based on beat-to-beat arterial pressure waveform. Using PRAM, we examined the temporal trends and adverse factors, in clinical management, of these performances.

**Methods:** We monitored blood pressure, cardiac index, cardiac cycle efficiency (CCE), dP/dT_max_, and systematic vascular resistance index in 91 children (aged 186 ± 256 days) during their first 48 h after cardiopulmonary bypass. Above parameters, inotropic and vasoactive drug dosages, and serum lactate were recorded 3-hourly. NT-proBNP was measured daily.

**Results:** CCE and dP/dT_max_ gradually increased (*P*s < 0.0001), while systematic vascular resistance index, diastolic blood pressure and inotrope dosages decreased (*P*s < 0.0001) over time. Cardiac index, systolic blood pressure, and heart rate did not change significantly (*P*s ≥ 0.231). Patients undergoing deep hypothermic circulatory arrest had significantly higher heart rate and lower CCE (*P*s ≤ 0.006) over time. Multivariate analyses indicated that epinephrine dose significantly correlated with systolic blood pressure, cardiac index, CCE, and dP/dT_max_ after polynomial transformation, with the peak ranging from 0.075 to 0.097.

**Conclusions:** Systemic hemodynamic and myocardial performance gradually improved in the first 48 h after cardiopulmonary bypass without the “classic” nadir at 9–12 h. Deep hypothermic circulatory arrest and higher epinephrine doses were adversely associated with these performances. CCE, rather than cardiac index or other common-used parameters, was the most sensitive and consistent indicator.

## Introduction

Hemodynamic monitoring is the definite cornerstone of the postoperative management in children with congenital heart disease (CHD) undergoing cardiopulmonary bypass surgery (CPB) (1). The early post-CPB period is characterized by myocardial injury, reduced cardiac output, and hemodynamic instability (1), requiring prompt and adequate adjustment of treatments, e.g., inotropic and vasoactive drugs. Cardiac output-guided hemodynamic therapy algorithm has been shown to improve outcomes in adults following cardiac surgery (2). No such study has been conducted in post-CPB children due largely to the technical limitations to directly monitor hemodynamics in young children and infants. The risk factors of postoperative hemodynamic and myocardial performance remain largely to be explored.

Since the invention of the Fick principle in 1870 (3), there have been intermittent technological developments to measure cardiac output, namely, Swan-Ganz catheter and thermodilution method in the 1970's (4), respiratory mass spectrometer in the 1990's (5–7). Thermodilution has well-known limitations when applied to post-CPB children (8). The presence of intracardiac shunt and tricuspid and pulmonary regurgitation can significantly affect its accuracy. Additionally, repeated cold saline injections are also undesirable in the fragile condition of cardiac function after CPB. It has been rarely used in children after CPB (1). Respiratory mass spectrometry is a desirable technique to measure oxygen consumption. Combined with arterial and venous blood gases, it is able to derive almost all elements of hemodynamics in varied circulations (5–7, 9). However, it is technically and timely highly demanding and hardly used outside of clinical research settings (8).

More recently, there have been several systems developed to directly monitor cardiac output and other hemodynamic parameters based on the arterial pulse contour analysis methods such as PiCCO2 and MostCare, etc (10). They are minimally invasive and in a real-time fashion, which is fundamental in post-CPB children. PiCCO2 combines pulse contour method with thermodilution for calibration, thereby inheriting the limitations (3). MostCare (Vygon Vytech, Padova, Italy) uses the pressure recording analytical method (PRAM) without the need for calibration (11, 12). It has been validated against the Fick method in pediatric patients (13) and used in children during CPB (14, 15), not yet in post-CPB children.

By using PRAM, we aimed to examine the temporal trends of systemic hemodynamic and myocardial performance parameters of children with CHD during the first 48 post-CPB hours, and more importantly to identify adverse factors of these parameters in routine clinical management.

## Patients and Methods

### Patients

After ethics approval (No. 46201), 91 children with CHD undergoing CPB were enrolled from July 2019 to June 2020. The informed consent was waived since PRAM monitoring by MostCare is our clinical routine. More complex CHD patients were selected daily when MostCare devices were available. Patients with other congenital anomalies or previous CPB were excluded.

### Intraoperative Procedures

Standard procedures were performed (16). DHCA (time ranged 15–26 min, median 18 min) was used in 17 patients to repair the coarctation of the aorta with ventricular septal defect or interruption of the aortic arch.

### Postoperative Management

Patients receive time-cycled pressure control/pressure support ventilation on arrival in the CICU, followed by non-invasive ventilation when appropriate. Ventilation settings are adjusted to maintain a PaCO_2_ at 35–45 mmHg and a SaO_2_ between 70 and 100% according to palliative or complete repair. The rectal temperature is maintained at 36–37°C. Sedation consists of continuous intravenous infusion of sufentanil (0.2 μg/kg/min) and dexmedetomidine (2 μg/kg/min) and intermittent injections of midazolam (0.1 mg/kg). Hematocrit is maintained at 34–45%. Inotropic and vasoactive drugs include dopamine, milrinone, and epinephrine to maintain arterial blood pressure (systolic pressure 60–90 and 90–105 mmHg and diastolic pressure of 40–60 and 55–66 mmHg in neonates and children, respectively). Despite the protocol, management varied among clinicians.

### Methods of Measurements

#### Direct Monitoring of Systemic Hemodynamic Parameters Using PRAM

A MostCare device based on PRAM was used to monitor patient during their first 48 postoperative hours, and data were collected 3-hourly. PRAM assesses systemic hemodynamics by analyzing frequently sampled (1,000 Hz) arterial pressure waveform. In any artery, volume changes with the radial expansion in response to pressure variations, which is mutually determined by left ventricle contraction, arterial elasticity, and backward pressure reflections (17). To estimate stroke volume, the whole pulsatile area under the systolic portion of the pressure curve is measured. Arterial impedance is determined from both systolic and diastolic phases of the pressure waveform, hence no calibration or preloaded data is necessary (17). PRAM measures heart rate, systolic blood pressure (SBP), diastolic blood pressure (DBP), cardiac index (CI), systemic vascular resistance index (SVRI), the maximal slope of systolic upstroke (dP/dT_max_), and cardiac cycle efficiency (CCE) (11). dP/dT_max_ indicates the maximal rate of the rise of arterial pressure manifesting left ventricular pressure and is used as an index of myocardial contractility (11). CCE is uniquely derived by PRAM and calculated according to the ratio of systolic energetic performance to the total energetic expenditure of the heartbeat using the following formula (18):

(1) $$\begin{array}{l} \frac{W{\left(t\right)}_{sys}}{W{\left(t\right)}_{beat}}*K\left(t\right) \end{array}$$

W(*t*)_*sys*_ is the power of the systolic stage of the heartbeat, W(*t*)_*beat*_ is the power of the whole cycle of the heartbeat, and K(*t*) is the ratio of mean pressure expected and mean pressure measured.

CCE evaluates the compensating interplay of different cardiovascular system compartments, i.e., left and right ventricular contractility, pre- and after-load, heart rate, reflected waves, as well as the elasticity of great arteries and the ventricular-arterial coupling (18). It ranges from negative values to +1. The negative values indicate the activation of *in vivo* compensation mechanisms supporting compartments of the cardiovascular system that do not work properly, while a higher CCE indicates a better overall cardiovascular functioning (18).

#### Clinical Data

Demographics, STAT categories (19), operative data, and clinical outcomes were recorded (Table 1). Doses of inotropic and vasoactive drugs and serum lactate were recorded 3-hourly. NT-proBNP was measured daily.

**Table 1:** Demographic and clinical data in 91 children undergoing cardiopulmonary bypass surgery.

**Variable**	**Number (%) or median (range)**
**Gender**	
Male	51 (56%)
Female	40 (44%)
**Weight, kg**	4.8 (2–12.5)
**Height, cm**	58 (41–98)
**Age, day**	104 (3–1,428)
**Prematurity**	14 (15.4%)
**STAT category**	
1	15 (16.5%)
2	32 (35.2%)
3	21 (23.1%)
4	19 (20.9%)
**Preoperative mechanical ventilation use**	15 (16.5%)
**Surgery time, h**	3.2 (1.6–10.4)
**Cardiopulmonary bypass time, min**	110 (34–429)
**Aortic cross-clamp time, min**	55 (0–205)
**Deep hypothermia circulatory arrest use**	17 (18.7%)
**Postoperative mechanical ventilation time, h**	48 (0–260)
**CICU stay, day**	4 (1–14)
**Death**	2 (2.2%)

### Statistical Analysis

Data were described as mean ± SD, median (range), or frequency (%) when appropriate. Mixed linear regression for repeated measures was used to (1) analyze the temporal trend of variables; (2) compare the difference between DHCA group and non-DHCA group in levels and trends with the analysis of the group effect and interaction between time and group. The parameter estimate and *P*-value of the group effect (*P*_group_) indicate the difference in the level of each measure between groups. Those of the time and group interaction (*P*_group × time_) indicate the difference in the trend of each measure between groups; (3) analyze correlations between measures. The fixed predictors were clinical variables, and outcomes were hemodynamic and myocardial variables. Polynomial transformation of the drug dose was tested regarding the best fit for the drug dose, in which the parameter estimate and *P*-value of dose (parameter estimate_dose_ and *P*_dose_) indicated lower doses, those for time^2^ or dose^2^ indicated higher doses. The peak value in the polynomial trend was determined by the following equation: X_peak_ = parameter estimate_dose_/(-2^*^${\text{parameter estimate}}_{\text{dose}}^{2}$). All analyses were performed with SAS 9.4.

## Results

Table 1 presents the demographic and clinical data of the cohort.

### Temporal Trends of Hemodynamics and Inotropic and Vasoactive Doses

**Figure 1:**
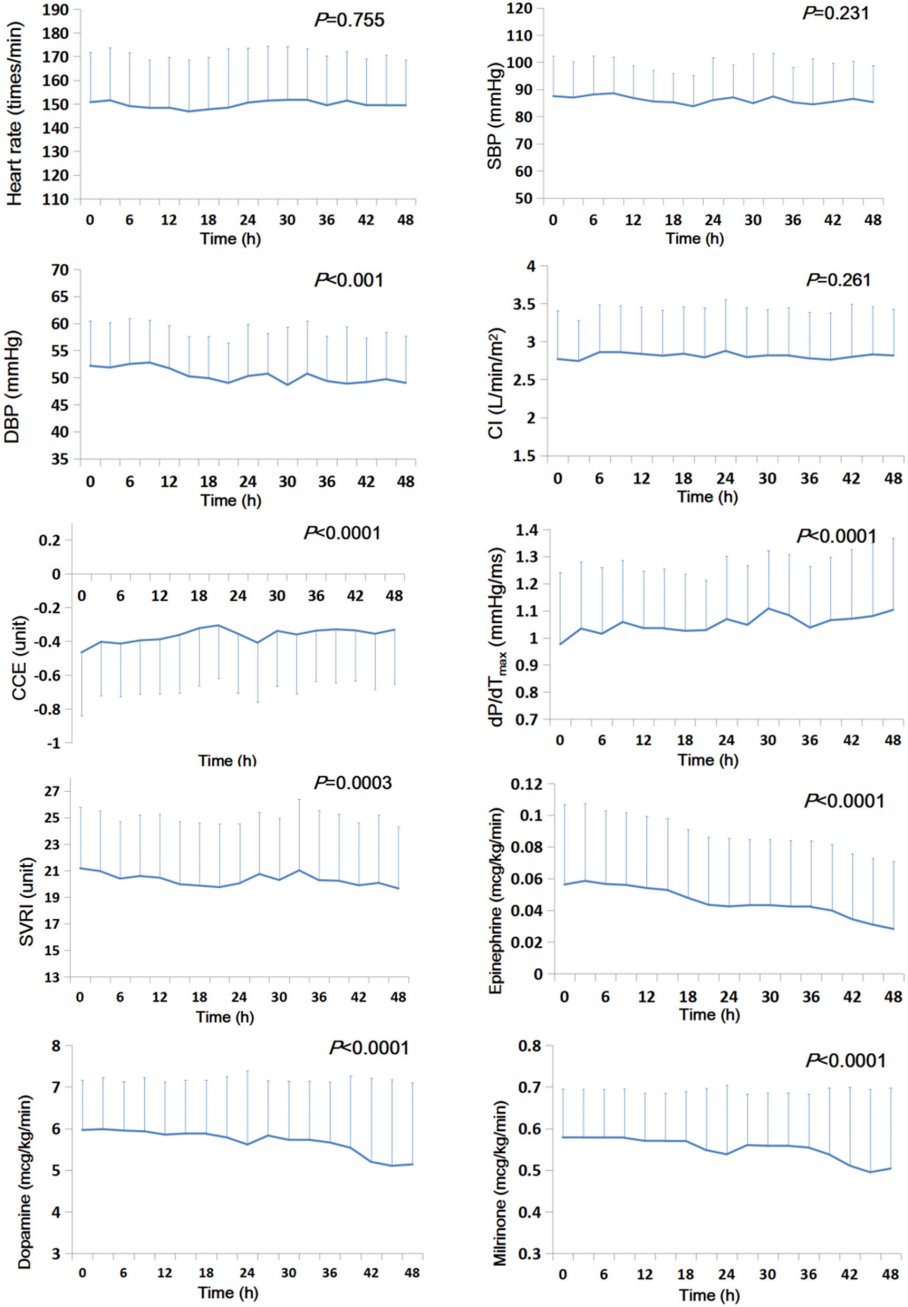
Temporal trends of systemic hemodynamic and myocardial status and doses of inotropic and vasoactive drugs during the first 48 h after CPB (*n* = 91). The mean value is presented, and the error bar represents the standard deviation. CCE, cardiac cycle efficiency; CI, cardiac index; DBP, diastolic blood pressure; dP/dT_max_, the maximal slope of systolic upstroke; SBP, systolic blood pressure; SVRI, systematic vascular resistance.

During the first 48 post-CPB hours, CCE and dP/dT_max_ gradually and significantly increased (*P*s < 0.0001), while SVRI and DBP decreased (*P*s ≤ 0.0003). The doses of epinephrine, dopamine, and milrinone decreased significantly (*P*s < 0.0001). CI, SBP, and heart rate did not change significantly (*P*s ≥ 0.231) (Figure 1 and Supplementary Table 1).

**Figure 2:**
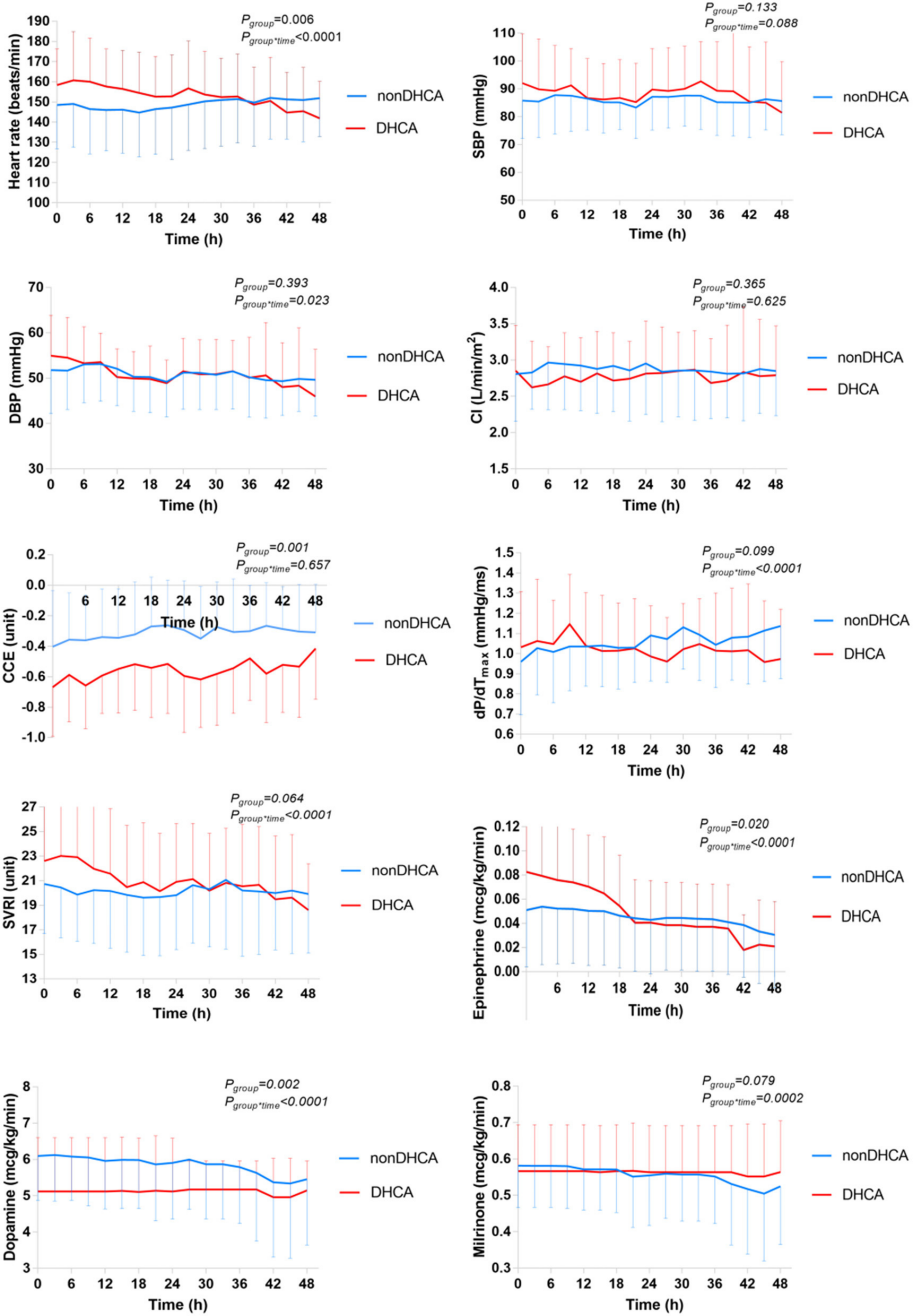
Comparison of the temporal trends of the systemic hemodynamic and myocardial status between DHCA (*n* = 17) and non-DHCA groups (*n* = 74) during the first 48 h after CPB. The mean value is presented, and the error bar represents the standard deviation. CCE, cardiac cycle efficiency; CI, cardiac index; DBP, diastolic blood pressure; dP/dT_max_, the maximal slope of systolic upstroke; SBP, systolic blood pressure; SVRI, systematic vascular resistance.

Patients undergoing DHCA (*n* = 17), compared with those without (*n* = 74), had or trended to have significantly higher heart rate (*P*_group_ = 0.006) and SVRI (*P*_group_ = 0.064), and lower CCE (*P*_group_ = 0.001) and dP/dT_max_ (*P*_group_ = 0.099) over time. They received significantly higher epinephrine (*P*_group_ = 0.020) and lower dopamine (*P*_group_ = 0.002), but were not different in milrinone (*P*_group_ = 0.479) (Figure 2 and Supplementary Table 2). After adjusted for the doses of inotropic drugs, the differences remained significant in heart rate (*P*_group_ = 0.031) and CCE (*P*_group_ = 0.006), but not in dP/dT_max_ (*P*_group_ = 0.395) and SVRI (*P*_group_ = 0.208) (Supplementary Table 1). CI did not differ between groups either including inotropic and vasoactive drug doses or not (*P* > 0.280).

### Correlations Between Clinical and Hemodynamic Variables

#### Univariate Analysis

As presented in Table 2, after adjusted for time, epinephrine dose significantly and positively correlated with heart rate, SBP, DBP, SVRI, dP/dT_max_, lactate, and NT-proBNP (*P*s ≤ 0.002). It significantly correlated with CCE after polynomial transformation, being positive at lower doses <0.083 mcg/kg/min (parameter estimate _dose_ = 2.63 *P*_dose_ <0.0001), and negative at higher doses (${\text{parameter estimate}}_{\text{dose}}^{2}$ = −15.88, $P_{\text{dose}}^{2}$ = 0.001). Dopamine dose significantly and positively correlated with CCE (*P* < 0.0001), negatively correlated with SVRI and NT-proBNP (*P*s ≤ 0.020). Milrinone dose significantly and positively correlated with CI (*P* = 0.033), negatively correlated with SVRI and lactate (*P*s ≤ 0.031). When tested as the dependent variable, epinephrine dose significantly and positively correlated STAT category and CPB time (parameter estimate = 0.01 and *P* = 0.016 for STAT; parameter estimate = 0.0002 and *P* = 0.016 for CPB time).

**Table 2:** Univariate and multivariate analyses of the correlations between inotropic and vasoactive drug doses and hemodynamic parameters in 91 children during the first 48 h after cardiopulmonary bypass.

**Variable**	**Heart rate**		**SBP**		**DBP**		**CI**		**CCE**		**dP/dTmax**		**SVRI**		**NT-proBNP**		**Lactate**	
	**Parameter estimate**	***P-*value**	**Parameter estimate**	***P*-value**	**Parameter estimate**	***P-*value**	**Parameter estimate**	***P*-value**	**Parameter estimate**	***P*-value**	**Parameter estimate**	***P-*value**	**Parameter estimate**	***P*-value**	**Parameter estimate**	***P*-value**	**Parameter estimate**	***P*-value**
**Univariate analysis**																		
Age	−0.03	0.001	0.01	0.0004	0.01	0.002	0.0002	0.433	0.0003	0.003	−0.00004	0.548	0.002	0.187	−11.57	0.001	−0.0004	0.402
STAT category	5.05	0.009	−1.13	0.224	−0.96	0.112	−0.07	0.245	−0.05	0.080	0.01	0.504	0.01	0.989	3332	<0.0001	0.11	0.479
CPB time	0.01	0.808	0.02	0.165	0.02	0.033	0.001	0.252	−0.001	0.169	3.165E−7	0.999	0.002	0.793	7.18	0.582	0.004	0.060
ACC time	0.07	0.184	−0.01	0.835	0.01	0.616	0.001	0.575	−0.001	0.244	−0.00004	0.936	−0.003	0.825	17.16	0.476	0.009	0.020
DHCA	5.67	0.261	2.14	0.370	−0.001	0.999	−0.11	0.424	−0.25	0.0002	−0.04	0.358	0.97	0.386	5208	0.017	−0.02	0.954
Epinephrine	193.05	<0.0001	37.57	0.002	28.40	0.0001	dose 2.56 dose^2^ −13.77	0.031 0.097	dose 2.63 dose^2^ −15.88	<0.0001 0.001	1.51	<0.0001	16.24	<0.0001	79094	<0.0001	5.29	0.001
Dopamine	−0.14	0.710	−0.34	0.237	−0.23	0.199	0.02	0.147	0.03	< .0001	−0.24	0.298	−0.25	0.001	−1194	0.020	−0.06	0.164
Milrinone	1.53	0.722	−1.10	0.748	1.28	0.542	0.298	0.033	0.11	0.156	−0.08	0.228	−2.04	0.024	−9488	0.158	−0.99	0.031
**Multivariate analysis**																		
Age	−0.02	0.014	0.02	0.001					0.0004	0.006								
CPB time									−0.001	0.036								
Epinephrine	202.22	<0.0001	dose 125.19 dose^2^ −806.87	<0.0001 0.001	27.02	0.001	dose 3.46 dose^2^ −22.95	0.015 0.041	dose 2.86 dose^2^ −14.70	0.0002 0.015	dose 3.17 dose^2^ −17.03	<0.0001 0.001	14.89	<0.0001	59,754	<0.0001	5.41	0.003
Dopamine									0.03	0.010								
Time	0.14	<0.0001			−0.06	<0.0001			0.004	<0.0001	0.003	<0.0001	−0.01	0.022	94.09	0.002	−0.03	<0.0001

*P-values < 0.05 were underlined.*

*ACC, aortic crossclamp time; CCE, cardiac cycle efficiency; CI, cardiac index; CPB, cardiopulmonary bypass; DBP, diastolic blood pressure; DHCA, deep hypothermic circulatory arrest; dP/dT_max_, the maximal slope of systolic upstroke; SBP, systolic blood pressure; SVRI, systematic vascular resistance index*.

Additionally, CCE significantly correlated with NT-proBNP (parameter estimate = −9072.59 and *P* < 0.0001) but not with lactate (*P* = 0.627). No significant correlations were found between CI and NT-proBNP (*P* = 0.764) or lactate (*P* = 0.721).

#### Multivariate Analysis

As presented in Table 2, epinephrine dose remained significantly correlated with CCE after polynomial transformation (${\text{parameter estimate}}_{\text{dose}}^{2}$ =2.86 and −14.70, respectively, *P*s ≤ 0.015 for both). Its correlations with other variables were different from the linear ones shown in the univariate analyses. After polynomial transformation, epinephrine dose significantly correlated with SBP (${\text{parameter estimate}}_{\text{dose}}^{2}$ = 125.19 and −806.87, respectively, *P*s ≤ 0.001 for both), CI (${\text{parameter estimate}}_{\text{dose}}^{2}$ = 3.46 and −22.95, respectively, *Ps* ≤ 0.041 for both), and dP/dT_max_ (${\text{parameter estimate}}_{\text{dose}}^{2}$ = 3.17 and −17.03, respectively, *Ps* ≤ 0.001 for both). SBP, CI, CCE, and dP/dT_max_ peaked at an epinephrine dose of 0.078, 0.075, 0.097, and 0.093 mcg/kg/min, respectively. Epinephrine dose linearly and positively correlated with heart rate, DBP, SVRI, lactate, and NT-proBNP (*P*s ≤ 0.003). Dopamine dose positively correlated with CCE (*P* = 0.010). Age significantly and negatively correlated with heart rate, and positively with SBP and CCE (*P*s ≤ 0.014). CPB time significantly and negatively correlated with CCE (*P* = 0.036). No significant correlation was found between other clinical and hemodynamic variables (*P*s ≥ 0.074).

## Discussion

By monitoring children with CHD during the first 48 post-CPB hours using PRAM, we found the followings: (1) systemic hemodynamics and myocardial status gradually improved without the “classic” nadir at 9–12 h; (2) patients undergoing DHCA had significantly higher heart rate and lower CCE; (3) higher epinephrine doses were adversely associated with overall systemic hemodynamics and myocardial performance, indicated by lower SBP, CI, dP/dT_max_ and CCE, and higher heart rate, DBP, SVRI, lactate, and NT-proBNP; (4) CCE was the most sensitive and consistent indicator among all PRAM parameters.

### Temporal Trends of Gradual Improvement of Hemodynamics

Wernovsky et al. in 1995 compared the postoperative hemodynamic profile in infants undergoing the arterial switch operation using low-flow CPB or DHCA (1). No significant between-group difference was found in blood pressure, CI, and SVRI measured by the thermodilution technique. They reported that, in both groups, CI reached a nadir, while SVRI reached a peak, at 9–12 h postoperatively and returned to baselines 24 h after surgery. They attributed the nadir to ischemic/reperfusion and systemic inflammatory response. This temporal trend has been widely used to guide clinical management. However, data from the present study showed a gradual improvement in overall systemic hemodynamics and myocardial status during the 48-h postoperative period, as indicated by the increase in CCE and dP/dT_max_ and the decrease in SVRI and inotropic and vasoactive drug doses; CI did not change significantly and no other parameters showed the nadir. This profile is consistent with our other studies that used more advanced monitoring techniques, such as respiratory mass spectrometry to oxygen consumption combined with the Fick principle in the past two decades, in which CI gradually increased over 72 h in post-CPB children including neonates following the Norwood procedure and infants with less complex CHDs following complete repair (5, 6). Other than these studies, few have addressed the temporal trend of CI in post-CPB children (20). The difference between the previous study (1) and ours may be due to a couple of reasons as follows. First, our cohort included a heterogeneous group of less complex CHDs with a lower proportion of neonatal surgery (18.7%) compared to the previous study in a homogeneous group of neonates with transposition of the great arteries undergoing arterial switch operation (1). Second, intraoperative management has improved significantly in the later era, e.g., the application of modified ultrafiltration (21), which reduces the systemic inflammatory response and improves ventricular function and oxygen transport (5, 22). The gradual hemodynamic improvement coincides with the reciprocal profiles of circulating cytokines and C-reactive protein (5). Such hemodynamic profiles should be kept in mind when managing post-CPB children in the current era.

### DHCA Was Associated With Inferior Myocardial Status

Another interesting finding was the inferior myocardial status, indicated by lower CCE and higher heart rate and epinephrine doses, among DHCA patients compared to those undergoing CPB (Figure 2). Neither perfusion technique perfused the heart (with lungs). Thus, this difference may be attributed to the difference between systemic and multi-organ ischemia/reperfusion caused by DHCA and that of a single organ by CPB (23). Correspondingly, rats undergoing DHCA had greater multi-organ vascular endothelial dysfunction and myocardial apoptosis as compared to CPB (24). Intestinal ischemia/reperfusion may lead to local and systemic inflammatory responses mediated by intestinal mast cells (25). Besides, deeper hypothermia used in DHCA may also contribute to the difference (26), as a result of greater myocardial excitation-contraction coupling disruption (27).

### Higher Doses of Epinephrine Were Adversely Associated With Hemodynamics

Epinephrine is widely used in children after CPB (20), with a wide dose range from 0.01 to 0.3 mcg/kg/min, and usually <0.1 mcg/kg/min (28). However, few studies evaluated its hemodynamic effects in children following CPB. Our data revealed that epinephrine was associated with a favorable hemodynamic and myocardial performance at lower doses (<0.08–0.1 mcg/kg/min), indicated by higher SBP, CCE, dP/dT_max_, and CI; at higher doses, these effects became adverse and counterintuitive.

The causal relationship between epinephrine and hemodynamics remains to be clarified, and it is further confounded by the fact that clinicians tend to use higher epinephrine doses in sicker patients, which is also the case in our patients. The favorable effects of low doses shown in our data are consistent with the finding in post-CPB adults. Stepwise increases of epinephrine from 0.02 to 0.08 mcg/kg/min linearly increased CI and decreased SVRI (29). When a wider dose range was tested in anesthetized piglets, the curvilinear effect was observed (30). Several mechanisms may underlie these observations. β-adrenergic overstimulation may cause tachycardia and cardiac dysfunction impairing cardiac filling and contractility (31). At the same time, α-adrenergic overstimulation may cause peripheral (32) and coronary arterial vasoconstriction (33), leading to increased SVRI and cardiac afterload, and reduced myocardial oxygen delivery (34). The latter may be further compounded by tachycardia result from increased myocardial oxygen consumption.

Both dopamine and milrinone doses positively correlated with hemodynamic and myocardial status. We previously reported that dopamine at 5 μg/kg/min in neonates after the Norwood procedure (STAT category 5) increased systemic and myocardial oxygen consumption but failed to increase CI and oxygen delivery, therefore exerting an adverse effect on systemic and myocardial oxygen transport (7). The patient population in this study is distinct from the previous study, with much less complex CHD (STAT category 1–4) and older age ranging 47–208 days (median 104), and they may have more cardiac function reserve and less stimulated increase in oxygen consumption due to less brown fat tissue (35). The beneficial effects of milrinone have been well-documented (36).

### CCE Was the Most Sensitive and Consistent Indicator to Reflect Hemodynamic Status

Finally, CCE significantly and positively correlated with NT-proBNP, while CI did not, consistent with previous findings in adults (12, 37). Taken together, CCE, rather than CI or other parameters, was most consistent and sensitive to reflect the hemodynamic effects of the clinical factors examined in this study. While CI is the final cardiac outcome from the complex interactions among the endogenous cardiac and neurohumoral responses and the exogenous inotropic and vasoactive drugs (38), CCE reflects the cost of cardiovascular work to maintain such a CI.

### Limitations

The observational nature of this study has several limitations. First, the causal relationship between epinephrine dose and hemodynamic outcomes showed by regression analysis remains speculative. Second, the epinephrine doses were relatively high and often combined with dopamine in our center. The situation is suboptimal but allowed us to reveal the curvilinear effect of epinephrine. The cut-off value might be different if not combined with dopamine or other inotropic and vasoactive drugs. This might also be applicable to the effect of dopamine if used along. Third, our cohort included a heterogeneous group of less complex CHDs with a lower proportion of neonatal surgery compared to contemporary patient populations from the advanced centers. A larger multi-center cohort study, including neonatal surgeries and complex cases and using more advanced techniques, is warranted to confirm our findings, in particular to the temporal trends of hemodynamics.

## Conclusions

In CHD children early after CPB, systemic hemodynamic and myocardial performance improved gradually during the first 48 h without the “classic” nadir at 9–12 h. These performances were worse in patients undergoing DHCA and adversely associated with higher doses of epinephrine >0.08–0.10 mcg/kg/min. CCE, rather than CI or other parameters, was most consistent and sensitive to reflect patient's hemodynamic status.

## Data Availability Statement

The raw data supporting the conclusions of this article will be made available by the authors, without undue reservation.

## Ethics Statement

The studies involving human participants were reviewed and approved by Guangzhou Women and Children's Hospital. Written informed consent from the participants' legal guardian/next of kin was not required to participate in this study in accordance with the national legislation and the institutional requirements.

## Author Contributions

XL and YL has contributed to the conducting and reporting of the work described in the article. YC, JianL, LL, LM, and MZ has contributed to the conducting of the work. XC has contributed to the planning and conducting of the research. JiaL has contributed to the planning, conducting, and reporting of the research. All authors contributed to the article and approved the submitted version.

## Conflict of Interest

The authors declare that the research was conducted in the absence of any commercial or financial relationships that could be construed as a potential conflict of interest.
