# Supplementary material for: Contemporary Trends and Risk Factors of Hemodynamic and Myocardial Mechanics Derived by the Pressure Recording Analytical Method After Pediatric Cardiopulmonary Bypass
Source: Front Cardiovasc Med. 2021 Jul 20;8:687150. doi: 10.3389/fcvm.2021.687150 (PMC8330813; doi:10.3389/fcvm.2021.687150)
Supplement: Supplementary file 1 [file Data_Sheet_1.docx]

**Supplemental Material**

**Supplemental Table 1. Mean±SD or median (range) values of the 3-hourly systemic hemodynamics and doses of inotropic and vasoactive drugs in 91 children during the first 48 hours after CPB**

| **Time**, h | **0** | **3** | **6** | **9** | **12** | **15** | **18** | **21** | **24** | **27** | **30** | **33** | **36** | **39** | **42** | **45** | **48** |
| --- | --- | --- | --- | --- | --- | --- | --- | --- | --- | --- | --- | --- | --- | --- | --- | --- | --- |
| **Epinephrine**, mcg/kg/min | 0.05  (0-0.22) | 0.05  (0-0.17) | 0.05  (0-0.17) | 0.05  (0-0.17) | 0.05  (0-0.17) | 0.05  (0-0.17) | 0.05  (0-0.16) | 0.05  (0-0.16) | 0.03  (0-0.16) | 0.05  (0-0.16) | 0.05  (0-0.16) | 0.04  (0-0.16) | 0.04  (0-0.16) | 0.03  (0-0.16) | 0.03  (0-0.16) | 0  (0-0.16) | 0  (0-0.16) |
| **Dopamine**, mcg/kg/min | 5.89  (3.70-9.00) | 6.00  (3.1-9.00) | 6.00 (3.10-9.00) | 6.00  (0-9) | 5.88  (0-9) | 5.90  (0-9) | 5.88  (0-9) | 5.78  (0-9) | 5.78  (0-9) | 5.71  (0-9) | 5.62  (0-9) | 5.62  (0-9) | 5.60  (0-9) | 5.60  (0-9) | 5.50  (0-9) | 5.4  (0-9) | 5.40  (0-9) |
| **Milrinone**, mcg/kg/min | 0.56 (0.33-0.96) | 0.56 (0.33-0.96) | 0.56 (0.33-0.96) | 0.56 (0.26-0.96) | 0.56 (0.26-0.86) | 0.56 (0.26-0.86) | 0.57 (0.13-0.86) | 0.56  (0-0.86) | 0.56  (0-0.86) | 0.56  (0-0.86) | 0.56  (0-0.86) | 0.56  (0-0.86) | 0.56  (0-0.86) | 0.54  (0-0.86) | 0.54  (0-0.86) | 0.53  (0-0.86) | 0.53  (0-0.86) |
| **Heart rate**, beats/min | 150.53±21.56 | 151.25±22.39 | 149.08±22.65 | 148.35±20.44 | 148.19±21.42 | 146.64±21.88 | 147.65±22.08 | 148.18±24.87 | 150.38±22.97 | 151.03±22.90 | 151.24±22.09 | 151.67±21.44 | 149.46±20.90 | 151.73±20.53 | 149.91±19.80 | 149.78±20.88 | 149.76±19.14 |
| **SBP**, mmHg | 79.44±28.46 | 83.48±20.80 | 85.16±21.19 | 82.49±25.33 | 81.80±22.92 | 82.60±19.00 | 81.73±20.43 | 78.15±23.71 | 79.98±27.68 | 84.44±20.25 | 79.70±28.37 | 83.42±24.44 | 84.04±18.60 | 80.88±24.76 | 77.96±27.41 | 79.78±26.19 | 75.63±29.47 |
| **DBP**, mmHg | 47.81±17.47 | 50.50±12.66 | 51.34±12.57 | 49.70±15.29 | 48.88±14.08 | 48.60±11.52 | 47.95±12.80 | 45.93±14.16 | 46.76±16.42 | 49.30±12.31 | 45.93±16.64 | 48.46±14.51 | 49.00±11.59 | 46.84±14.75 | 44.94±15.72 | 45.91±15.37 | 43.58±17.29 |
| **CI**, L/min/m^2^ | 2.82±0.64 | 2.79±0.54 | 2.91±0.64 | 2.91±0.62 | 2.88±0.62 | 2.87±0.60 | 2.88±0.63 | 2.84±0.67 | 2.89±0.77 | 2.83±0.67 | 2.78±0.76 | 2.82±0.73 | 2.81±0.64 | 2.76±0.71 | 2.82±0.70 | 2.86±0.65 | 2.84±0.62 |
| **CCE**, unit | -0.47±0.37 | -0.40±0.32 | -0.41±0.31 | -0.39±0.32 | -0.39±0.33 | -0.36±0.35 | -0.32±0.34 | -0.31±0.31 | -0.35±0.35 | -0.41±0.35 | -0.34±0.33 | -0.36±0.35 | -0.34±0.30 | -0.33±0.32 | -0.34±0.30 | -0.36±0.33 | -0.33±0.32 |
| **dP/dT_max_**, mmHg/ms | 0.98±0.27 | 1.03±0.25 | 1.01±0.25 | 1.06±0.23 | 1.04±0.21 | 1.03±0.22 | 1.03±0.21 | 1.03±0.19 | 1.07±0.23 | 1.05±0.22 | 1.11±0.22 | 1.08±0.22 | 1.04±0.23 | 1.07±0.23 | 1.07±0.26 | 1.08±0.27 | 1.1±0.26 |
| **SVRI**, unit | 21.17±4.61 | 20.96±4.54 | 20.4±4.31 | 20.59±4.62 | 20.46±4.80 | 19.97±4.72 | 19.86±4.74 | 19.75±4.74 | 20.04±4.49 | 20.74±4.65 | 20.29±4.67 | 21.01±5.37 | 20.27±5.26 | 20.23±5.03 | 19.89±4.70 | 20.07±5.09 | 19.65±4.62 |

*CCE,* cardiac cycle efficiency; *CI,* cardiac index *; DBP*, diastolic blood pressure; *dP/dT_max_,* maximal left ventricular pressure rise rate; *SBP*, systolic blood pressure; *SVRI*, systematic vascular resistance index

**Supplemental Table 2. Effect of deep hypothermic circulatory arrest (DHCA) on the temporal trends of hemodynamic variables and doses of vasoactive and inotropic drugs**

| Variable | Heart rate | |  | SBP | |  | DBP | |  | CI | |  | CCE | |  | dP/dTmax | |  | SVRI | |  | Epinephrine | |  | Dopamine | |  | Milrinone | |
| --- | --- | --- | --- | --- | --- | --- | --- | --- | --- | --- | --- | --- | --- | --- | --- | --- | --- | --- | --- | --- | --- | --- | --- | --- | --- | --- | --- | --- | --- |
|  | Parameter estimate | *P* value |  | Parameter estimate | *P* value |  | Parameter estimate | *P* value |  | Parameter estimate | *P* value |  | Parameter estimate | *P* value |  | Parameter estimate | *P* value |  | Parameter estimate | *P* value |  | Parameter estimate | *P* value |  | Parameter estimate | *P* value |  | Parameter estimate | *P* value |
| **Analysis of groups** | | | | | | | | | | | | | | | | | | | | | | | | | | | | | |
| Time | 0.07 | 0.002 |  | -0.01 | 0.807 |  | -0.06 | <0.0001 |  | -0.001 | 0.222 |  | 0.003 | <0.0001 |  | 0.003 | <0.0001 |  | -0.007 | 0.204 |  | -0.001 | <0.0001 |  | -0.02 | <0.0001 |  | -0.002 | <0.0001 |
| DHCAgroup | 14.56 | 0.006 |  | 3.94 | 0.133 |  | 1.45 | 0.393 |  | -0.14 | 0.365 |  | -0.24 | 0.001 |  | 0.08 | 0.099 |  | 2.14 | 0.064 |  | 0.03 | 0.020 |  | -1.10 | 0.002 |  | -0.02 | 0.479 |
| DHCAgroup  *Time | -0.38 | <0.0001 |  | -0.08 | 0.088 |  | -0.06 | 0.023 |  | 0.001 | 0.625 |  | -0.0005 | 0.657 |  | -0.005 | <0.0001 |  | -0.05 | <0.0001 |  | -0.001 | <0.0001 |  | 0.02 | <0.0001 |  | 0.001 | 0.0002 |
| **Analysis of groups and inotropic drugs** | | | | | | | | | | | | | | | | | | | | | | | | | | | | | |
| Time | 0.18 | <0.0001 |  | 0.01 | 0.667 |  | -0.05 | 0.001 |  | -0.0001 | 0.933 |  | 0.004 | <0.0001 |  | 0.003 | <0.0001 |  | -0.003 | 0.570 |  |  |  |  |  |  |  |  |  |
| DHCAgroup | 9.97 | 0.031 |  | 2.43 | 0.374 |  | 0.11 | 0.950 |  | -0.16 | 0.280 |  | -0.21 | 0.006 |  | 0.04 | 0.395 |  | 1.49 | 0.208 |  |  |  |  |  |  |  |  |  |
| DHCAgroup  *Time | -0.24 | <0.0001 |  | -0.03 | 0.504 |  | -0.03 | 0.343 |  | -0.002 | 0.404 |  | -0.001 | 0.469 |  | -0.004 | <0.0001 |  | -0.03 | 0.010 |  |  |  |  |  |  |  |  |  |
| Epinephrine | 176.07 | <0.0001 |  | 34.22 | 0.006 |  | 26.07 | 0.001 |  | 0.91 | 0.092 |  | 0.55 | 0.059 |  | 1.29 | <0.0001 |  | 13.42 | 0.0001 |  |  |  |  |  |  |  |  |  |
| Dopamine | -0.34 | 0.501 |  | -0.51 | 0.219 |  | -0.63 | 0.014 |  | -0.01 | 0.740 |  | 0.04 | <0.0001 |  | -0.001 | 0.931 |  | -0.24 | 0.035 |  |  |  |  |  |  |  |  |  |
| Milrinone | 8.10 | 0.176 |  | 3.30 | 0.499 |  | 6.71 | 0.025 |  | 0.34 | 0.098 |  | -0.23 | 0.043 |  | -0.05 | 0.603 |  | 0.49 | 0.714 |  |  |  |  |  |  |  |  |  |

*P* values <0.05 were underlined. *CCE,* cardiac cycle efficiency; *CI,* cardiac index; *DBP*, diastolic blood pressure; *DHCA*, deep hypothermic circulatory arrest; *dP/dT_max_*, maximal left ventricular pressure rise rate; *NT-proBNP*, N-terminal pro-B-type natriuretic peptide; *SBP*, systolic blood pressure; *SVRI*, systematic vascular resistance index
